# Two novel connexin32 mutations cause early onset X-linked Charcot-Marie-Tooth disease

**DOI:** 10.1186/1471-2377-7-19

**Published:** 2007-07-09

**Authors:** Geir J Braathen, Jette C Sand, Geir Bukholm, Michael B Russell

**Affiliations:** 1 Faculty Division Akershus University Hospital, University of Oslo, 1474 Nordbyhagen, Oslo, Norway; 2 Institute for clinical epidemiology and molecular biology (Epi-Gen), Akershus University Hospital, 1478 Lørenskog, Oslo, Norway; 3 Department of Laboratory Medicine, Genetic section, Telemark Hospital, 3710 Skien, Norway; 4 Department of Neurology, Akershus University Hospital, 1478 Lørenskog, Oslo, Norway; 5 Department of Research and Development, Akershus University Hospital, 1478 Lørenskog, Oslo, Norway

## Abstract

**Background:**

X-linked Charcot-Marie Tooth (CMT) is caused by mutations in the connexin32 gene that encodes a polypeptide which is arranged in hexameric array and form gap junctions.

**Methods:**

We describe two novel mutations in the connexin32 gene in two Norwegian families.

**Results:**

Family 1 had a c.225delG (R75fsX83) which causes a frameshift and premature stop codon at position 247. This probably results in a shorter non-functional protein structure. Affected individuals had an early age at onset usually in the first decade. The symptoms were more severe in men than women. All had severe muscle weakness in the legs. Several abortions were observed in this family. Family 2 had a c.536 G>A (C179Y) transition which causes a change of the highly conserved cysteine residue, i.e. disruption of at least one of three disulfide bridges. The mean age at onset was in the first decade. Muscle wasting was severe and correlated with muscle weakness in legs. The men and one woman also had symptom from their hands.

The neuropathy is demyelinating and the nerve conduction velocities were in the intermediate range (25–49 m/s). Affected individuals had symmetrical clinical findings, while the neurophysiology revealed minor asymmetrical findings in nerve conduction velocity in 6 of 10 affected individuals.

**Conclusion:**

The two novel mutations in the connexin32 gene are more severe than the majority of previously described mutations possibly due to the severe structural change of the gap junction they encode.

## Background

Charcot-Marie-Tooth (CMT) disease is the most common inherited disorder of the peripheral nervous system with an estimated prevalence of 1 in 2,500 [1]. It is clinically, neurophysiologically and genetically a heterogeneous disorder. Clinical features are distal muscle wasting and weakness, sensory loss with reduced tendon reflexes and foot deformities [2,3]. Neurophysiology can classify CMT into type 1 and 2, depending on whether the motor conduction velocity (MCV) is less or above 38 m/s [4]. A third form has intermediate MCV [5]. CMT is inherited as an autosomal dominant, autosomal recessive or X-linked disorder [6]. It has been estimated that autosomal dominant inheritance accounts for 88%, X-linked for 9% and autosomal recessive for 3% of the inherited forms of CMT [1]. The majority of CMT cases are caused by a duplication of peripheral myelin protein 22 (PMP22), which is carried by 71% of those with CMT 1 [7]. X-linked CMT caused by mutations in the connexin32 gene (GJB1) is probably the 2^nd ^most frequent cause of CMT [8]. In humans there are at least 20 different connexin genes [9]. The polypeptides encoded by a connexin gene are arranged in hexameric array and form gap junctions [10]. Gap junctions contain hydrophilic membrane channels that allow direct communication between neighboring cells through the diffusion of ions, metabolites and small signaling molecules [11]. Mutations in connexin genes can beside CMT cause the skin disorders Vohwinkel syndrome and erythrokeratoderma variabilis, cardiovascular disorders, cataract and deafness [12-17]. Connexin32 is expressed in both the peripheral and central nervous system (PNS and CNS). It is expressed in the paranodal region and incisures of myelinating Schwann cells, and in cell bodies and processes of oligodendrocytes, while it is not expressed in the compact myelin layer [18]. This is interesting since some patients with mutations in the connexin32 gene have hearing loss, subclinical CNS symptoms, acute or chronic clinical CNS manifestations as well as CNS involvement on brain MRI [19-22].

We present two novel mutations in the Connexin32 gene and the clinical features in the two families.

## Methods

### Patients

Families from Norway with possible X-linked Charcot-Marie-Tooth disease were analyzed for mutations in the connexin32 gene. The two families were from west and southeast of Norway.

### Examination

**Figure 1:**
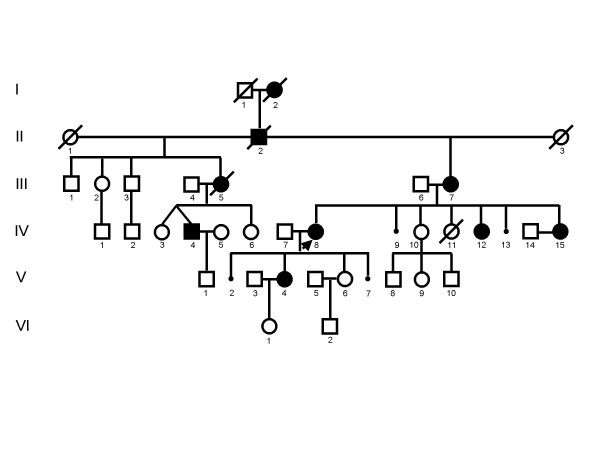
**shows a pedigree with novel mutations in connexin32**. III-7, IV-4, IV-8, IV-12, IV-15 had the mutation c.225delG (R75fsX83) while IV-10, IV-14 and V-6 did not have the deletion.

Affected and unaffected family members available were interviewed and clinically examined by geneticist and neurologist GJB. Information on III-1, III-2, III-3, III-4, III-6, IV-1, IV-2, IV-3, IV-5, IV-6, VI-1 and VI-2 from family 1 was derived from the proband, IV-8 (Fig. 1A). In family 2 information on II-2, II-4 and II-5, the latter representing 3 unaffected siblings whom had no affected children was based on the proband, IV-1 and his mother III-2 (Fig. 1B). The interview was semi-structured and the participants received a thorough genetic and neurological examination. All findings were recorded in an evaluated Norwegian scheme designed for neuropathy with additional items added by GJB [23-25].

### Neurophysiology

GJB conducted the majority of the neurophysiological examinations. The patients were acclimatized to room temperature 25°C prior to the neurophysiological examination. Standard techniques with surface electrodes were used for antidromic sensory conduction and orthodromic motor conduction. Needle electrodes were used, if the surface electrodes failed to record conduction velocities of the sural nerve. In general both the right and left sural, superficial peroneal and tibial nerves were examined and some had their median nerves examined too. Electromyography (EMG) was performed in left leg muscles; abductor hallucis, extensor digitorum brevis, peroneus longus, tibialis anterior and posterior, and gastrocnemius. Only gene carriers had a neurophysiological examination once.

### Genetics

DNA was extracted from leucocytes using QIAgen FlexiGene kit. The coding region of the connexin32 gene was amplified using Invitrogen Platinum taq polymerase Cat.Nos 10966-026 and 2 mM MgCl_2_. Primers used was forward AATGAGGCAGGATGAACTGG and reverse CCTGGTATGTGGCATCAGC spanning the exon 2 creating an 877 bp band. Annealing temperature was 63°C and number of cycles was 35. The sequencing was carried out using internal forward primer TGCTCTACCCTGGCTATGC starting at base 589 and reverse primer CCACATTGAGGATGATGCAG starting at base 745 and in some cases reverse primer GCCATGCACGTGGCTCACCAGCAA starting at base 297 in addition to the PCR primers and BigDye Terminator kit version 1.1 Applied Biosystems. Sequences were analyzed on an ABI3100 automated DNA sequencer (Applied Biosystems). Numbering of nucleotides is according to the open reading frame of the cDNA sequence, GenBank accession NM_000166s[26].

The karyotype was analyzed using standard G-band technique of the chromosomes.

## Ethics

The study was approved by the Regional Committees for Medical Research Ethics.

The patients gave written consent for participating in the study.

## Results

**Figure 2:**
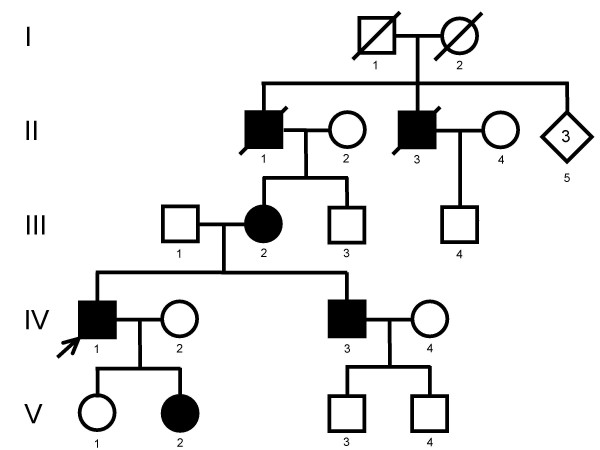
shows a pedigree with novel mutations in connexin32. III-2, IV-1, IV-3, V-1 and V-2 had the mutation c.536G>A (C179Y) while IV-2 and IV-4 did not have the G→A transition.

Fig. 1 and 2 show two pedigrees with novel mutations in connexin32. Family 1 had a c.225delG (R75fsX83) which causes a frameshift and premature stop codon at position 247. The original sequence from amino acid 76 was changed from LWSLQLILV.. to CGPCSSSX. Family 2 had a c.536G>A (C179Y) transition which causes a change of cysteine to tyrosine at position 179.

### Family 1

This pedigree is compatible with X-linked inheritance. III-2 is shown as an unaffected individual, since the only information we have is that she had had a transient psychiatric problem during teenage. She also had psychiatric problems and later committed suicide. Affected female descendants of II-2 and II-3 have only female descendants and spontaneous abortions of unknown gender as well as a stillborn daughter (IV-11), while unaffected females have descendants of both gender.

### Family 2

This pedigree is compatible with X-linked inheritance, although I-2 is shown as an unaffected individual, since we had no information about her. V-1 carries the mutation, but was unaffected at age 8 years.

### Age at onset

Women had a mean age at onset at 8.3 years (range 1–22), and men had a mean age at onset at 6 years (range 3–11). Table 1 shows that the first symptoms usually were muscle cramps and weakness, but problems skiing and skating were also often encountered as the first symptom.

**Table 1:** First symptom of Charcot-Marie-Tooth disease.

*Family member*	*Sex*	*Age at onset years*	*First symptom*
Family 1			
III-7	♀	6	Muscle cramps, thin legs, problems going skiing and skating
IV-4	♂	3	Muscle cramps, problems with running and reduced balance
IV-8	♀	10	Problems with running and skating and reduced balance
IV-12	♀	6	Problems with running and excessive falling
IV-15	♀	22	Excessive tiredness
V-4	♀	7	Problems with running and skiing
			
Family 2			
III-2	♀	6	Muscle cramps, problems with running and reduced balance
IV-1	♂	4	Weakness in lower extremities, problems with running and walking and reduced balance
IV-3	♂	11	Weakness in lower extremities, problems with running and walking and reduced balance
V-1	♀	1	Reduced balance and problems with walking

### Clinical characteristics

Table 2 and Table 3 show the clinical characteristics of CMT. Men were more severely affected than women, and also had a faster disease progression.

### Motor symptoms

Muscle wasting was correlated with muscle weakness and the symptoms were symmetrical. All men had severe muscle weakness in their legs and hands, while only one woman had marked symptoms from her hands.

### Sensory symptoms

Affected men and three affected women had reduced pain sensation. Sense of vibration was reduced in all but one. Sensation of proprioception and touch was generally preserved.

### Tendon reflexes

The Achilles reflex was reduced or absent in all affected individuals, while two women and all affected men had reduced or absent reflexes in the upper extremities.

### Deformities

The majority had hammertoes and pes cavus, while two men also had excavated palms.

### Ataxia

One man had ataxia.

### Balance

Balance problems were experienced by the majority and this symptom was more pronounced in family 2 than in family 1.

**Table 2:** Clinical characteristics of Charcot-Marie-Tooth scored according to Neuropathy Impairment score (NIS) unless otherwise indicated.

	Family 1						Family 2			
Family member	III-7	IV-4	IV-8	IV-12	IV-15	V-4	III-2^1^	IV-1	IV-3	V-2

Sex	♀	♂	♀	♀	♀	♀	♀	♂	♂	♀
Age at onset	6	3	10	6	22	7	6	4	11	1
Disease duration	62	41	36	30	7	13	51	33	25	6
*Clinical characteristics*										
Muscle wasting^NIS^										
Underarm	1	1	1	1	0	1	0	2	1	0
Hand	1	2	2	1	0	1	1	2	2	1
Thigh	1	1	0	1	0	0	0	1	0	0
Leg	1	2	2	2	0	2	1	2	2	0
Feet	1	2	2	2	1	2	2	2	2	0
Muscle weakness^NIS^										
Elbow flexion	0	0,5	0	0	0	0	1	0	0	0
Hand flexion	0	0,5	0	0	0	0	0	0,5	1	0
Hand extension	0	0,5	0	0	0	0	0	0,5	0	0
Finger flexion	0,5	1	0,5	0,5	0	0	0	0,5	0	0
Finger spreading	0,5	1	0,5	0,5	0	0,5	0,5	1,5	2	0
Thumb, abduction	0,5	2	1	0,5	0	0,5	0,5	5	2	0
Knee flexion	0	0	0	0	0	0	0	0,5	0	0
Ankle dorsi-Flexion	0,5	2	1	1/0,5^2^	0	0,5	0,5	2	3	0
Ankle plantar-Flexion	0	1,5	0,5	1	0	0	0	2	1,5	0
Toe extensor	1	2	1	1,5	0,5	1	0,5	2	3	0
Toe flexor	1	2	0,5	0,5	0	0,5	0,5	2	1,5	0
Sensory loss										
*Touch*^3^										
Thigh	0	0	0	0	0	0	1	0	0	0
Feet, leg	0	0	0	0	0	0	1	0	0	0
*Pain*^3^										
Hand, underarm	0	1	0/1^2^	1	0	0	1	1	1	0
Thigh	0	1	0	1	0	0	1	1	0	0
Feet, leg	0	1	1	1	0	0	2	2	1	0
*Vibration*^3^										
Hand	0	1	1	0	0	0	2	2	1	0
Knee	1	2	1	1	0	0	2	2	1	0
Ankle	2	2	2	2	1	0	2	2	1	0
1. metatarsal	2	2	2	2	2	2	2	2	2	0
1. toe	2	2	2	2	2	2	2	2	2	0
*Proprioceptive*^NIS^										
Toe	0	2	0	0	0	0	1/0^2^	0	0	0
Reflexes^NIS^										
Biceps	0	1	0	2	0	0	0	2	2	1
Triceps	0	0	0	1	0	0	0	1	0	0
Brachioradialis	0	1	0	1	0	0	0	1	0	0
Patellar	0	1	0	2	0	1	1	2	2	1
Achilles	2	2	2	2	2	2	1	2	2	1
Deformities^3^										
Excavated palms	0	0	0	0	0	0	0	2	1	0
Pes cavus	1	2	2	0	1	2	1	2	2	0
Hammertoes	1	2	2	1	0	1	1	0	2	0
Syndactyly	0	0	0	0	0	0	0	0	1	0
Kyphoscoliosis	0	1	0	0	0	1	0	0	0	0
Ataxia in legs^3^	0	0	0	0	0	0	0	1	0	0
Romberg^3^	1	1	1	1	0	0	0	2	2	1

^1^She had a right cerebral infarction at age 52 years and a slight spasticity of the left arm as sequelae.

^2^Asymmetrical signs right/left.

^3^0 = normal; 1 = mild/moderate affected modality; 2 = severe affected modality.

**Table 3:** Neuropathy Impairment Score (NIS) of the Charcot-Marie-Tooth patients in the two families.

	Family 1						Family 2			
Family member	III-7	IV-4	IV-8	IV-12	IV-15	V-4	III-2	IV-1	IV-3	V-2

Sex	♀	♂	♀	♀	♀	♀	♀	♂	♂	♀
Age at onset	6	3	10	6	22	7	6	4	11	1
Disease duration	62	41	36	30	7	13	51	33	25	6
NIS										
Sum cranial nerves	0	0,5	0	0	0	0	0	0	0	0
Muscle weakness										
*MRC-sumscore*^1^	1	5	2	2	0	1	3	5	8	0
*Sum UE*^2^	3	10	4	3	0	2	4	16	10	0
*Sum LE*^3^	5	15	6	7,5	1	4	3	17	18	0
Sum muscle weakness^4^	8	25	10	10,5	1	6	7	33	28	0
Sum reflex deficit	2	10	4	16	4	6	2	16	12	6
Sum sensory deficit	4	14	6	8	4	4	15	14	10	0
Total sum NIS^5^	14	49,5	20	34,5	9	16	24	63	50	6

^1^MRC sum score; 6 muscles on both sides, 3 muscle upper extremity (UE) and 3 muscles lower extremity (LE) respectively.

^2^Sum UE 9 muscles R and L.

^3^Sum LE 8 muscles R and L.

^4^Sum muscle weakness 19 muscles.

^5^Total sum NIS: The score consists of Sum cranial nerves, Sum muscle weakness, Sum reflex deficit and Sum sensory deficit

**Table 4:** Electroneurographical findings in affected, gray shading indicate men. Sensory nerve action potential (SNAP) (μV), compound muscle action potential (CMAP) (mV)

*Family member*	*Sex*	*Age at onset (yr)*	*Age at examination (yr)*	*Sensory Sural nerve*				*Motor Peroneal nerve*				*Motor Tibial nerve*				*Sensory Median nerve*				*Motor Median nerve*				*EMG Chronic denervation*
				*SNAP*		*CV*		*CMAP*		*CV*		*CMAP*		*CV*		*SNAP*		*CV*		*CMAP/*		*CV*		
Family 1				*R*	*L*	*R*	*L*	*R*	*L*	*R*	*L*	*R*	*L*	*R*	*L*	*R*	*L*	*R*	*L*	*R*	*L*	*R*	*L*	
III-7	♀	6	68	-	-	-	-	3.3	1.7	43.8	37.0	-	-	-	-									Present
IV-4	♂	3	44	-	-	-	-	-	-	-	-	-	-	-	-							33.5	36.6	Present
IV-8	♀	10	46	-	-	-	-	1	0.2	32.8	25.4	0.8	1.7	30.2	31.1	-	-	-	-	1.0	0.3	36.8	34.6	Present
IV-12	♀	6	36	-	0.6	-	30.4	-	-	-	-	1.2	1.3	25.2	25.0									Present
IV-15	♀	22	29	-	-	-	-	5.5	4.0	39.5	41.9	4.0	5.8	44.1	46.9									Slightly
V-4	♀	7	20	-		-	29.3	-	-	31.8	31.7	-	-	29.7	32.5									Present
																								
Family 2																								
III-2	♀	6	44	-	-			1.5		29.0		0.5		30.0						2.5		44.0		Present
IV-1	♂	4	37	-	-	-	-	-	-	-	-	-	-	-	-	-	-	-	-	0.3	0.2	25.8	22.7	Present
IV-3	♂	11	18							31.0						-		-				30.0*		Present
V-1	♀		8	3.8		57,8		0.8	1.3	45.9	45.8													Not present
V-2	♀	1	7	1.0		48.3		2.0	1.3	48.8	46.7													Not present

- not recordable

* ulnar nerve

### Neurophysiological and neuroradiological investigations

Table 4 shows the neurophysiological findings. The amplitudes and distal motor latencies were in accordance with the delayed conduction velocities. Both motor and sensory nerve conduction velocities were reduced or not obtainable in both the lower and upper extremities. The seven years old girl (family 2, V 2) had asymmetrical CMAP amplitudes and motor conduction velocities in the low-normal range in the lower extremities. The conduction velocities were more reduced in men than in women. The majority of conduction velocities were symmetrical, but most of the affected family members had one asymmetrical finding. All EMG findings were consistent with peripheral neuropathy except in the seven years old girl. A man had a visual evoked potentials (VEP) investigation showing severe delayed latency on both sides, while a magnetic resonance imaging (MRI) of his brain taken some years later was normal. He had no visual disturbances.

## Discussion

Our main finding is the identification of two novel mutations in connexin32. Family 1 had a c.225delG (R75fsX83) which cause a frameshift and a premature stop at position 247. This causes an altered transmembrane (M2) portion and a shortened polypeptide which is lacking two of the four transmembrane domains (M3 and M4), an extracellular loop (E2), the intracellular loop and the C-terminal end [27]. The exact mechanism of connexin32 channel assembly is not known to our knowledge. We hypothesize that the polypeptide encoded by the mutated connexin32 gene result in a polypeptide which is not arranged in a hexameric array due to the change in protein structure. Connexin32 is expressed in early human placenta as well as placenta tissue culture [28,29]. The human placenta differs from other somatic tissues, since it has an ability to reverse the X-inactivation program [30]. Thus, it is likely that female fetuses with the connexin32 mutation will have only normal connexin32 gap junction in the placenta, while male fetuses with the mutation will lack connexin32 gap junctions. Family 1 is characterized by at least four spontaneous abortions. We could not explain this by chromosome abnormalities as III-7 has a normal female karyotype 46,XX. Other etiologies to recurrent spontaneous abortions are anatomical, endocrine, placenta abnormalities, infection, smoking and alcohol consumption, other environmental factor, psychological trauma and stressful life event, certain coagulation and immunoregulatory protein defects [31]. We have no knowledge that any of these causes are of significance in this family. However, increased rate of abortions has not been described in other CMTX families. Furthermore connexin26 and connexin43 are expressed in the placenta, and may compensate for the possible loss of connexin32 function [32]. III-7 and IV-8 have seven female and no male offspring an incidence that happens by chance in 1/128. We have no explanation for the spontaneous abortions in this family.

Family 2 had a point mutation in the connexin32 gene, c.536G>A (C179Y). This mutation causes an amino acid change from cysteine to tyrosine at position 179, which is the last cysteine residue in the E2 loop. The E1 and E2 loops each have three highly conserved cysteine residues [27]. Thus, the number of disulfide-bridges stabilizing the loops between two hemi-channels from two opposed cell are reduced, which alter the function of the connexin32 gap junction. One man had ataxia in the lower extremities. This is likely to be a symptom of peripheral nature due to the sensory disturbances although a central origin can not be excluded. Balance problems were prominent in both families irrespective of disease duration and disease severity, while the neurological examination revealed normal proprioception in nearly all affected individuals. Thus, balance problems might represent a CNS symptom, since balance is based on vision, proprioception and equilibrium. The literature describes central symptoms such as deafness, extensor plantar response, transient ataxia, dysarthria, dysphagia or weakness as well as abnormal VEP, brainstem auditory evoked potentials and central motor evoked potentials [22,33-35]. Brain MRI shows transient, recurrent, white matter lesions in X-linked Charcot-Marie-Tooth disease [33,34]. One man had pathological visual evoked potential (VEP) with delayed latency on both sides, but a normal MRI of the brain taken some years later. All affected individuals carried the mutation and unaffected individuals did not carry it with one exception. An obligate gene carrier had the mutation (family 2, V-1), but was clinically asymptomatic at age 8 years, while she had reduced CMAP neurophysiologically. We defined age at onset as the first recorded clinical symptom that could be ascribed to CMT. The mean age at onset was in the first decade in both families. The mean age at onset was 4–12 years lower in men and 9–11 year lower in women than previously reported in X-linked CMT [36,37]. The mean age at onset in women will of course increase depending on the age at onset in the asymptomatic obligate gene carrier (family 2, V-1). Since eight women carried the gene, each 8 year period will increase the mean age at onset one year in women. Onset before 10 years was encountered in 27% of male and 15% of female in a study of 93 patients with X-linked CMT from 37 different families with 27 different connexin32 mutations [36]. Thus, the two novel mutations cause an earlier disease onset than the majority of the previously described connexin32 gene mutations. The literature reports of very early onset of X-linked CMT in a 15 months old girl with severe symptoms [36]. Our patient (family 2, V-2) had less severe symptoms with age at onset at 16 months. Men had in general more severe and faster disease progression than women, in accordance with the literature [4,22,36,38,39].

The neurophysiological examinations of affected individuals were compatible with intermediate type of CMT (25–49 m/s). Affected individuals had symmetrical clinical findings, while the neurophysiological examination revealed minor asymmetrical results in nerve conduction velocities. However, we only conducted the neurophysiological examination once, so the data are not reproduced. This is likely to be a real phenomenon, since the amplitudes and distal latencies (not shown in table 3) showed variation in accordance with the nerve conduction velocities. Neither X-inactivation alone nor the specific mutations can explain the asymmetry as it occurred in both men and women and in both families. Asymmetries in motor conduction velocities have also been reported by others [40,41]. Otherwise the neurophysiological findings were similar to those of five other studies of X-linked CMT [4,22,36,38,39].

## Conclusion

We conclude that the two novel mutations in the connexin32 gene have an earlier onset than the majority of previously described mutations.

## Competing interests

The author(s) declare that they have no competing interest.

## Authors' contributions

GJB acquired the material, conceived the study, participated in the design of the study and drafted the manuscript. JCS carried out the molecular genetic studies and the sequence alignment. GB participated in the design of the study and helped to draft the manuscript. MBR conceived the study, participated in the design of the study and drafted the manuscript. All authors read and approved the final manuscript.

## Pre-publication history

The pre-publication history for this paper can be accessed here:


